# Role of Tc99m sulfur colloid scintigraphy in differentiating non-cirrhotic portal fibrosis from cirrhosis liver

**DOI:** 10.4103/0972-3919.78247

**Published:** 2010

**Authors:** Dhritiman Chakraborty, Hejjaji Venkataramarao Sunil, Bhagwant Rai Mittal, Anish Bhattacharya, Baljinder Singh, Yogesh Chawla

**Affiliations:** Department of Nuclear Medicine, Postgraduate Institute of Medical Education and Research, Chandigarh, India; 1 Hepatology, Postgraduate Institute of Medical Education and Research, Chandigarh, India

**Keywords:** Liver cirrhosis, NCPF, scintigraphy, Tc99m sulfur colloid

## Abstract

**Background:**

Two most important causes of portal hypertension are cirrhosis of liver and non-cirrhotic portal fibrosis (NCPF). The purpose of this study was to assess the scintigraphic appearances of Tc99m sulfur colloid liver scan in differentiating liver cirrhosis from NCPF.

**Materials and Methods:**

Retrospective analysis records of 146 patients (91 male and 55 female) with diffuse hepatocellular disease was done for liver size, liver uptake, spleen size, spleen uptake, colloid shift to bone marrow and lungs.

**Methods:**

Retrospective analysis records of 146 patients (91 male and 55 female) with diffuse hepatocellular disease was done for liver size, liver uptake, spleen size, spleen uptake, colloid shift to bone marrow and lungs.

**Results:**

Cirrhotic livers showed patchy and lower uptake than NCPF (59% vs. 20%, *P*-value 0.041). Spleen size was significantly increased in NCPF compared to cirrhosis (100% vs. 67%, *P*-value 0.0137). Significant colloid shift to bone marrow was noted in cirrhosis (84% vs. 7%, *P*-value<0.0001).

**Conclusion:**

Tc99m sulfur colloid liver scan is a non-invasive procedure having a useful adjunctive role in clinical differentiation of cirrhosis from NCPF.

## INTRODUCTION

Cirrhosis is a consequence of chronic liver disease characterized by replacement of liver tissue by fibrosis, scar tissue and regenerative nodules leading to loss of liver function.[1,2] Cirrhosis is most commonly caused by chronic alcohol intake, hepatitis B and C and fatty liver disease but has many other possible causes. Some cases are idiopathic. It is diagnosed by clinical features, altered liver function, ultrasonography and liver biopsy. Non-cirrhotic portal hypertension (NCPH) comprises a group of diseases that are characterized by an increase in portal pressure due to intra-hepatic or pre-hepatic lesions, in the absence of cirrhosis of the liver. It is not merely absence of cirrhosis, but also of hepatic venous outflow obstruction, such as veno-occlusive disease and Budd-Chiari syndrome. The lesion in NCPH is generally vascular, present in the portal vein, its branches or in the peri-sinusoidal area of the liver. Wedged hepatic venous pressure (WHVP) is near normal or mildly elevated in these patients and is significantly lower than portal vein pressure.[3,4]

The majority of diseases that are grouped under this category of NCPH have PH as a late manifestation of the disease. The common causes of NCPH include schistosomiasis, non-cirrhotic portal fibrosis (NCPF), extra-hepatic portal vein obstruction (EHPVO), idiopathic portal hypertension (IPH), Budd-Chiari syndrome, veno-occlusive disease and congenital hepatic fibrosis. Two diseases, which are very common in developing countries and almost always present only with features of PH, include NCPF and EHPVO. Both NCPF and EHPVO could develop in a genetically predisposed individual when infection or a prothrombotic event could precipitate thrombosis in the portal vein or its radicals. If it is a major thrombotic event, occurring at an early age in life, the main portal vein becomes occluded, leading to development of EHPVO. However, in the event of repeated microthrombotic events, the small or medium branches of the portal vein are affected, leading to the development of NCPF in a young adult.[5]

Differentiation of NCPF and cirrhosis of liver is many times a clinical dilemma. The diagnosis of NCPF is established by the presence of features of PH like esophageal varices on endoscopy, raised splenic pulp pressure, collaterals on splenoportovenography (SPV) or on ultrasound), by a definite exclusion of cirrhosis of the liver on clinical, biochemical, ultrasonography, surgical and histological findings and by exclusion of obstruction of splenoportal axis on SPV and on Doppler ultrasound. Ultrasound findings that differentiated NCPF from cirrhosis included the absence of irregularities of intra-hepatic portal vein radicles, thickening of the portal vein wall ≥3 mm, a sudden narrowing of intra-hepatic second degree portal vein branches, and Gamna-Gandy bodies in the spleen and smooth surface of the liver.[6]

Liver biopsy is gold standard but it is invasive and a risky procedure in these patients with altered coagulation profile. Tc99m sulfur colloid has been used as an imaging agent for the liver and spleen.[7] Subjective assessment of parameters such as the dimensions of the liver and spleen, colloid shift and uptake of radiopharmaceutical in the bone marrow have been used for both diagnosis of liver cirrhosis and evaluation of its progression. The distribution of radio-colloid uptake in the liver, spleen and bone marrow has been shown to correlate well with the severity of chronic liver disease, the severity of histological fibrosis, prognosis and hepatic function.[8–11] This study was designed to assess the uptake patterns of Tc99m sulfur colloid in cirrhosis of liver and NCPF.

## MATERIALS AND METHODS

We retrospectively analyzed the studies of consecutive 146 (91 male, 55 female) patients of diffuse hepatocellular disease based on clinical and laboratory findings referred for Tc99 sulfur colloid liver scan. Age range was 8-78 years (mean age 36 years ± 14.6). All patients underwent Tc99m sulfur colloid liver scan 30 minutes after intravenous injection of 4mCi of the radiotracer. Eight [anterior, posterior, right anterior oblique, left anterior oblique, right posterior oblique, left posterior oblique, right lateral, left lateral] isocount (500000) planer images were acquired in 256×256 matrix under a dual head gamma camera equipped with low energy high-resolution collimator (E.Cam, Siemens, Erlangen, Germany). Images were visually interpreted for liver size and uptake, spleen size and uptake, and were graded as normal, increased or decreased in size or uptake with reference to normal scan. The sulfur colloid uptake in the bone marrow and in the lungs was evaluated as present or absent in all the scans.

### Statistical analysis

Values are expressed as mean±1 SD (95% confidence interval for the mean). Analysis of qualitative variables was done by non-parametric Mann-Whitney U-test. Differences were considered significant if the *P*-value was <0.05.

## RESULTS

**Figure 1:**
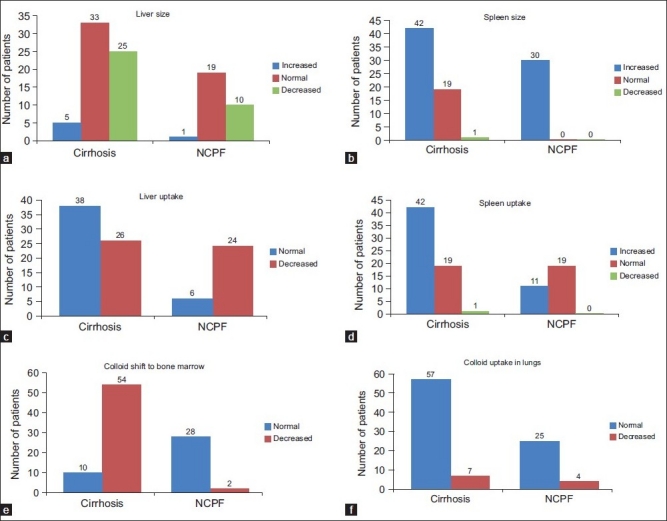
representing comparison of liver size (a), spleen size (b), liver uptake (c), spleen uptake (d), bone marrow uptake (e) and lung uptake (f) in patients with cirrhosis and NCPF (a-f respectively)

**Figure 2:**
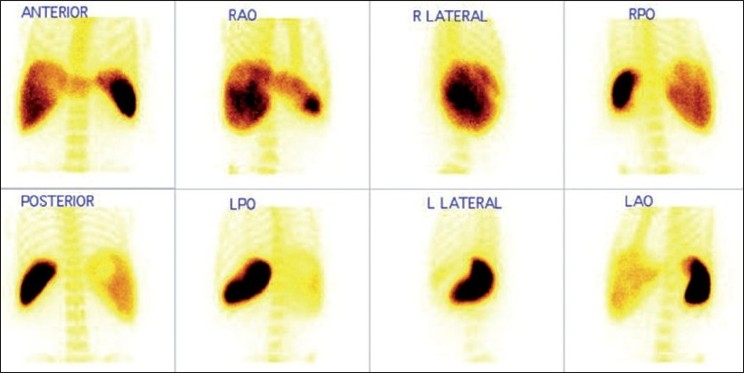
Tc99m sulfur colloid scan in case of cirrhosis of the liver showing impaired colloid uptake in the liver and increased uptake in the spleen with significant colloid shift to bone marrow

**Figure 3:**
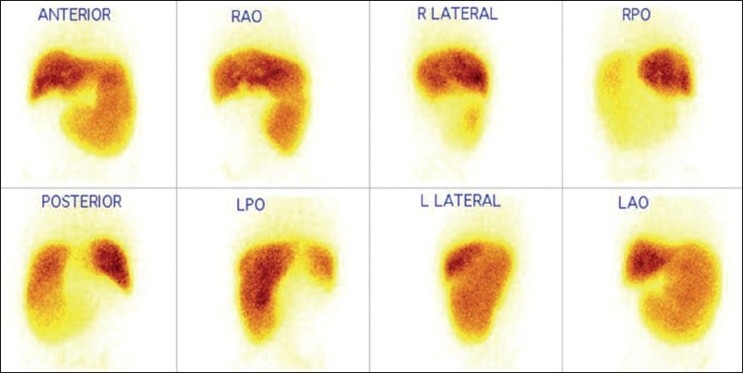
Tc99m sulfur colloid scan in case of NCPF showing adequate colloid uptake in liver with increased size and uptake in the spleen and no evidence of colloid shift to the bone marrow

Of the total 146 patients, 64 patients were diagnosed to have cirrhosis and 30 patients were of NCPF [Table 1]. Remaining 52 patients had normal scintigraphic findings. When analyzed for qualitative parameters mentioned above, no significant difference was found in liver size between cirrhosis and NCPF [Table 2]. Liver uptake was found to be normal in 41% compared to 80% in NCPF. Splenomegaly was noted in 100% NCPF patients compared to 67% in cirrhosis (*P*-value=0.0137). However, no significant difference was noted in splenic uptake in both the conditions. Significant colloid shift to bone marrow was seen in cirrhosis (89%), when in NCPF it was seen in only 7% (*P*-value=0.0001). Liver size, liver uptake, spleen size, spleen uptake and bone marrow uptake in patients with cirrhosis and NCPF are represented in Figure 1 (A-F respectively). Image 2 depicts scintigraphic findings in a patient with NCPF while image 3 represents findings in a patient diagnosed to have cirrhosis. [Figures 2 and 3]

**Table 1:** Qualitative scan findings in cirrhosis and NCPF

Parameters	Scan findings	Cirrhosis N=64	NCPF N= 30
Liver size	Increased	5 (8)	1 (3)
	Normal	33 (52)	19 (64)
	Decreased	26 (40)	10 (33)
Liver uptake	Decreased	38 (59)	6 (20)
	Normal	26 (41)	24 (80)
Spleen size	Decreased	1(2)	0
	Normal	19 (31)	0
	Increased	42 (67)	30 (100)
Spleen uptake	Decreased	1 (2)	0
	Normal	19 (31)	19 (63)
	Increased	42 (67)	11 (37)
Colloid shift to bone marrow	Absent	10 (16)	28 (93)
	Present	54 (84)	2 (7)
Colloid shift to lungs	Absent	57 (89)	26 (87)
	Present	7 (11)	4 (13)

Note: Splenic uptake was evaluated in 92 patients, as two had history of spelenectomy; Figures in parenthesis are in percentage

**Table 2:** Results of Mann-Whitney U test of the qualitative parameters

Parameters	*P*-value	Comment
Liver size	0.8425	No significant difference
Liver uptake	0.041	Significant difference (Lower in cirrhosis)
Spleen size	0.0137	Significant difference (Smaller in cirrhosis)
Spleen uptake	0.3950	No significant difference
Colloid shift to bone marrow	< 0.0001	Significant difference (Higher in cirrhosis)
Colloid uptake in lungs	0.7180	No significant difference

(*P* < 0.05 is significant)

## DISCUSSION

Hepatocytes and Kupffer cells are equally affected by the ﬁbrotic processin patients with liver cirrhosis.[8–10] Variable involvement of portal tracts known as obliterative porto-venopathy of liver is commonly seen in NCPF. NCPF is diagnosed by the presence of unequivocal evidence of PH in the definite absence of liver cirrhosis and EHPVO. The disease is characterized by massive splenomegaly with anemia, preserved liver function and benign prognosis in a majority of patients. Although differential diagnosis from liver cirrhosis is not always easy, liver histology, laparoscopy, portography, hepatic venography and measurement of WHVP have been shown to be useful in diagnosis. Relative distribution of radioactive colloid in cirrhotic livers using planar and SPECT techniques has been studied and reported previously.[8–10] They showed that the Kupffer cell mass determined by the degree of relative hepatic and splenic uptake of colloid particles by the cells of the reticuloendothelial system correlates with disease severity and hepatic function in liver cirrhosis. They also implied that radioactive colloid distribution is a better estimate of disease severity than invasive tests.[9] Thus, the uptake of radioactive colloid by the liver could be used to evaluate cirrhosis. Spleen volume undergoes modifications during the course of chronic liver disease. Measurement of spleen size and individual uptake of radio colloid by the spleen could be useful in evaluating spleen hyper function in patients with cirrhosis.

In our study colloid shift to the bone marrow (84 % vs. 7%), and decreased liver uptake (59% vs. 20%), was seen more often in cirrhotic group. Increased spleen size (100% vs. 67%) with no evidence of colloid shift to the bone marrow (84% vs. 7%), was more suggestive of NCPF. However, we could not compare our results with the gold standard histopathology for the final diagnosis. Increased bone marrow activity has been described in 16.6, 44. and 72.72% patients with Child A, B and C cirrhosis respectively.[11] Normally approximately 85% of the colloid is trapped in the kuffer cells in the liver and remainder goes mostly to spleen and bone marrow. Distinguishing pattern in cirrhosis is explained by cirrhotic liver’s decreased extraction efficiency for the blood-laden colloid due to pH, extra- and intra-hepatic arterio-venous shunt and replacement of the liver parenchyma and sinusoidal kuffer cells by fatty infiltration, necrosis and fibrosis. Thus colloid not extracted by the diseased liver recirculates through the bone marrow and spleen and is engulfed by reticuloendothelial cells of these areas, resulting in significant uptake in the bone and spleen.

In conclusion 99mTc sulfur colloid liver scan is a non-invasive procedure having a useful adjunctive role in clinical differentiation of cirrhosis from NCPF.
